# Exploring the association between personality and attitudes towards ageing in UK and Canadian older adults’: Use of a novel Behavioural Artificial Intelligence solution

**DOI:** 10.1371/journal.pone.0347422

**Published:** 2026-04-29

**Authors:** Stuart W. Flint, Eva Klein, Leonard Waverman, Ezra Goldberg, Mohammad Kaykanloo, Hager Ghouma, Stuart Sherman

**Affiliations:** 1 School of Psychology, University of Leeds, Leeds, United Kingdom; 2 Scaled Insights, Nexus, University of Leeds, Leeds, United Kingdom; 3 DeGroote School of Business, McMaster University, Ontario, Canada; University of West Florida, UNITED STATES OF AMERICA

## Abstract

Perceptions of ageing is an important psychosocial factor that influences health, wellbeing as well as engagement in health- promoting behaviours as people age. This study used a novel Behavioural Artificial Intelligence (AI) solution to examine the association between personality and attitudes towards ageing, and to explore how perceptions of ageing impact health promoting behaviours in UK and Canadian adults. Using a cross-sectional online survey methodology, 1011 UK and 1023 Canadian adults aged 65 years and older were recruited. Participants completed the attitudes towards ageing questionnaire short form and responded to 5 open-ended questions relating to their perceptions of ageing. Natural language computed in the open-ended responses was analysed using Scaled Insights Behavioural AI solution to examine personality attributes associated with attitudes towards ageing. Thematic analysis was also conducted to explore themes that emerged in the participants responses to the open-ended questions. Two personality clusters were identified that were associated with attitudes towards ageing.: Optimistic Ageing and Pessimistic Ageing. The Optimistic Ageing personality cluster had significantly more positive attitudes towards ageing; i.e., towards physical change, and psychosocial loss. Six significant themes emerged relating to participants perceptions of ageing, between those in the Optimistic Ageing and Pessimistic Ageing personality clusters; 1) attitudes towards physical activity and health, 2) mental and emotional health, 3) social connections and relationships, 4) independence and autonomy, 5) attitudes towards ageing and mortality, and 6) experiences of ageism. The findings highlight the important role of personality in attitudes towards ageing and the need for psychologists and other interventionists to consider personality in addressing negative attitudes towards ageing as well as actions to promote healthy behaviour. The novel Behavioural AI solution employed in this study, highlights the potential value of understanding personality both in predicting attitudes towards ageing but also in designing interventions and communications that are more personalised and target older adults based on their personality attributes.

## Introduction

Over the past 50 years, overall life expectancy has risen across the world, and this increase has been particularly evident in developed countries. Life expectancy is expected to continue to rise; the World Health Organization estimates that by 2050, there will be double (22% compared to 12% in 2015) the amount of people aged ≥ 60 years [1]. This increase is anticipated to be particularly pronounced in low- and middle-income countries [2]. Longer life expectancy has led to an increased ‘older adult’ population and consequently a rise in associated chronic health outcomes, mobility-related issues and associated social care costs [3]. Beard and Colleagues note that there is scarce evidence that increased longevity is accompanied by extended periods of good health [4]. Longer life expectancy, and thus, population ageing, has significant implications for healthcare, the labour market, pensions, housing and social services. This profound population shift highlights the need to foster healthy active ageing that enables people to continue to be part of and contribute to communities as they age.

Societal attitudes towards ageing are mostly negative, with stereotypes and stigma often attached to “getting older” and perceptions of older adults include general incompetence, lacking mental capacity, frailty, vulnerability and unattractiveness [5–6]. This can lead to experiences of bias and discrimination where older people are treated in an inequitable fashion including exclusion and marginalisation from settings such as the workplaces or communities. These experiences associated with reduced social functioning, isolation and a reduction in physical and mental health, wellbeing, less physical activity and a lower overall quality of life. Experiences of ageism, and internalisation of negative attitudes towards ageing increase the risk of negative health outcomes and chronic disease and behaviours often associated with ageing. The widespread nature of negative attitudes towards ageing and its impact has led to policies and strategies to improve active participation and contribution of older adults in communities [7]. The UK Government Office of Science highlights that attitudes towards ageing are a modifiable risk factor, i.e., they are affected more by societal and self-perceptions, idiosyncratic appraisals and emotions rather than the severity of physical symptoms of age-related conditions [8].

Self- Perceptions of ageing has consistently been reported as an important psychosocial factor that influences health and wellbeing as people age, particularly in older adult populations. Factors reported to influence self-perceptions of ageing include quality of life, mental and physical health, physical and social functioning, brain activity, individual differences and experiences of or expected ageism. A recent systematic review examining the association between self-perception of ageing and quality of life of older adults aged ≥ 60 years reported a strong association where quality of life was lower for older adults who had a negative self-perception of ageing. It was also reported that higher morale and good physical capability were associated with positive perceptions of health [6]. This is consistent with findings from Warmoth et al. [5] whose systematic review of observational studies focused on older adults’ own perceptions of health and functioning relating to ageing. They reported that in each of the seven domains examined more negative self-perceptions of ageing were associated with poorer health and functioning. The seven domains were health status, well-being and QOL, memory and cognitive performance, physical and physiological performance, medical conditions and outcomes, disability and functioning, healthy behaviours, and mortality. The relationship between individuals’ perceptions of ageing and engagement and maintenance of healthy behaviours such as physical activity have also been a consistent focus with studies reporting on the positive association between more positive own attitudes towards ageing with greater physical activity and health outcomes [9–10]. Encouraging positive perceptions of ageing and maintenance or adoption of physical activity is paramount with substantial evidence highlighting that such better self-perception can prevent or ameliorate chronic disease, prevent premature mortality, and enhance physical function and wellbeing.

Research has highlighted the need to remain autonomous and to have independence as a person ages. Losing independence is a key concern for many people as they age [11–12]. Given the importance of autonomy as a basic human need and independence as a person ages, there is a need to support people to live longer, healthier, independent lives where increased longevity is accompanied by extended periods of good health. This can have a significant effect on quality-adjusted life years (QALY; a measure of how many years of a person’s life they are in good health and wellbeing) [13].

Understanding the reasons for varied perceptions of ageing and subsequently providing appropriate support to older adults can reduce the health risks and behavioural responses often associated with ageing. Human behaviour is driven by a multitude of factors with personality a key driver that influences both behavioural adoption and change. Indeed, self-perceptions can be influenced and moderated by personality traits. Research has examined the association between personality and mental and physical health-related outcomes. For instance, personality traits have been identified as key factors in risk stratification for coronary artery disease, the leading cause of death in Western countries [14–15]. Much of the literature examining risk and indeed prognosis of cardiovascular disease in relation to personality have focused on Type D personality, which is characterized by a tendency towards emotional distress, with high scores of social inhibition and negative affective traits. Alternatively, traits including openness to experience and intelligence are associated with better general health, memory retention (for some openness facets) and physical functioning as people age [16–17]. Personality may provide key insights regarding the perceptions and approach that older adults have towards ageing, and as such, requires greater examination. To date, there is a dearth of AI driven approaches to examining personality and ageing. Thus, the current study aimed to explore the association between personality and attitudes towards ageing, as well as factors that impact attitudes towards ageing. The study aimed to achieve this by using Scaled Insights novel Behavioural Artificial Intelligence (AI) solution which assesses and subsequently clusters personality attributes associated with behaviours and outcomes. It was hypothesised that older adults’ attitudes towards ageing is associated with personality characteristics.

## Methods

### Design and sample

Using a cross-sectional design, 1011 UK and 1023 Canadian adults aged ≥ 65 years self-recruited to the study. The study was advertised to potential participants who had signed up to take part in research through Qualtrics^TM^ and Ask Canadian participant pooling mechanisms and had met the eligibility criteria. The study was granted ethical approval by the School of Psychology Research Ethics Committee at University of Leeds (REC number PSYC-891) and McMaster University Research Ethics Board (Reference Number 6591).

### Materials

A cross-sectional survey was developed comprising of closed (Attitudes to Ageing Questionnaire-Short Form, AAQ-SF) [18], as well as open-ended questions relating to perceptions of ageing and demographic questions.

Attitudes to Ageing Questionnaire Short Form (AAQ-SF):^18^ The AAQ-SF consists of 12-items, with 3 domains and 4 questions in each: Physical Change, Psychosocial Loss and Psychological growth reflecting both perceived positive and negative aspects of ageing. The AAQ-SF utilises a 5-point Likert scale for responses to each question (1 = strongly disagree, 5 = strongly agree), with total domain scores range between 4–20. The AAQ-SF has adequate internal consistency (α > .60) mirroring the structure of the original 24-item AAQ [19].

Open-ended questions: Five open-ended questions were comprised to gather rich, in-depth insights about participants perceptions of ageing and to gather sufficient natural language data to be analysed using Scaled Insights Behavioural AI solution. The questions all had a minimum response requirement of 50 words:

1. Describe how your approach to daily life might have changed as you have got older
2. Describe the factors that you think are important to living independently as you age
3. As you have gotten older, have your thoughts about yourself (e.g., physical condition, capability) changed, and if so, why?
4. What motivates or demotivates you to participate in physical activity?
5. To what extent do you think physical activity is related to your physical and/or mental health and why?

Demographics: Participant responded to questions about gender, age, ethnicity and whether participants have a long-term health condition, or a disability. Qualtrics survey platform automatically captures longitudinal and latitudinal data which was used to identify indices of multiple deprivation (IMD).

### Procedure

Between 11^th^ July 2024 and 17^th^ October 2024, older adults aged ≥ 65 years were recruited in the UK and Canada using participant pooling mechanisms Qualtrics^TM^ and AskingCanadians. The study, hosted on Qualtrics^TM^ was advertised to older adults who met the inclusion criteria and those that were interested in participating read the information sheet and informed consent form. Older adults who provided written consent, accessed the survey comprised of demographic questions, the AAQ-SF [18], and the open-ended questions. Survey completion ranged between 15–25 minutes.

### Data analysis

The data was imported into a Jupyter Notebook, where code was written using Python, using the pandas library for data analysis. Relevant libraries included numpy, sklearn for machine learning models, seaborn & matplotlib for data visualization, scipy for statistics.

First the data was imported, then went through a series of filtering. Filtering included removing incomplete surveys, in the selected age demographic (≥ 65 years), which provided a large enough text sample to be analysed by the Scaled Insights Behavioural AI solution, over 100 words were needed. Additionally, filtering included removing data from analysis if text spam was detected, this was done using the sklearn library for machine learning, and uses two metrics for text similarity, the Jaccard and the Cosine models. The text from each respondent’s series of open text answer questions was combined, to make one text sample. To extract features from text, TfidfVectorizer from sklearn was used, before both the Jaccard and Cosine metrics were applied to each text sample. The results of both text similarity models were averaged, and responses that scored above a similarity threshold were removed from the analysis.

Next, the Scaled Insights NLP tool was applied to the open text data, analysing text to score each respondent on 113 personality traits. The Scaled Insights NLP tool is a set of machine-learning models designed to estimate behavioural traits based on patterns in language. Instead of diagnosing individuals or assigning fixed labels, the system identifies probabilistic tendencies, for example, an increased likelihood of being more cautious. The models were trained on very large datasets in which people had provided both language samples and validated psychometric assessments of personality. By learning the statistical associations between word use, sentence structure, tone, and known psychological measures, the system can estimate 113 personality-related features. Each trait score reflects how similar a new text sample is to the language patterns of individuals who scored highly on that trait during model development. Outputs depend on writing quality, length, context, and situational tone. This is why filtering removed text responses of less than 100 words, since short text results in less reliable estimates. The system reflects patterns present in the training data, so results should be interpreted as model-based approximations rather than direct psychological evaluation.

A clustering analysis was done, with inputs being the 113 personality traits. Elbow plot and Silhouette plots were run, and 2 distinct clusters were found as the best fit for the data. The data was sorted by greatest difference, to find the distinguishing traits for each cluster. Data was visualized and communicated using a variety of Python libraries and Microsoft Excel charts.

To explore demographic variable analysis, data was filtered again to only include those who had provided voluntary demographic data. To estimate deprivation, IMD was calculated using the UK Government “English Indices of Deprivation 2019” [20], “Scottish Index for Multiple Deprivation 2020” [21], Welsh Index of Multiple Deprivation [22], and Northern Ireland Multiple Deprivation Measure 2017 [23]. IMD for the Canadian sample was calculated using the 2022 postal code conversation file (PCCF (v8B)); consensus geography for Canada [24]. As the Canadian IMD uses a quintile scale for four factors, and not the decile scale used for the UK IMDs, a method was developed to harmonize the scales for cross-regional comparisons. First, the scores across the four quintile factors were summed to create a combined score, which could range from 4 (lowest quintile across all factors) to 20 (highest quintile across all factors). This combined score was then mapped onto a decile scale by dividing the range into 10 equal intervals using linear binning. The resulting scores were categorized into deciles using these bins, with labels ranging from 1 (least deprived) to 10 (most deprived). This approach ensured consistency with the UK IMD decile scale while retaining the relative deprivation rankings inherent to the Canadian IMD data

Code was written to summarize categorical data (gender, ethnicity, existing long-term health condition, disability, IMD) and to analyse continuous data (age). Two-way ANOVA were conducted to examine differences based on personality cluster (i.e.,., Cluster = 2X2 for country, gender, long-term health condition, disability, 2X8 for ethnicity and 2X10 for IMD), and independent t-tests were conducted to examine differences based on participant age.

Statistical analyses were performed using Python (version 3.12.7), primarily using the library scipy and its related stats group of functions (version 1.14.1). Statistical significance was defined as *p* < 0.05, and data quality was assessed prior to analysis.

Qualitative data captured within the five open-ended questions were also analysed to explore themes that emerged in the data. Two of the authors (SWF, SS) independently coded 100 responses from each of the open-ended questions. SWF then coded the remaining questions using these codes and had regular meetings with SS to ensure there was consistency and agreement with the application of the coding. Where any inconsistencies, rationale for the labels were offered by the independent coders to establish if and why these occurred. In the minimal number of inconsistencies, these were primarily the code name provided by the coders. Statements summarising each theme were created for each question and thematic analysis used [25]. Coding disagreements were resolved through discussion between the authors and there was agreement on the final themes, subthemes and supporting quotes.

## Results

### Descriptive analysis

Descriptive results are presented in Table 1 for age, gender, ethnicity, long-term health condition status, disability status and IMD for the whole sample, separated by country of residence and based on two distinct personality clusters that were identified within the data see Supplemental materials for the percentage of participants based on level of deprivation in Canada and the UK (S1 Fig) and Indices of multiple deprivation by the two personality clusters (S2 Fig). Of the 2034 participants, 1938 responded to the demographic questions and are presented in Table 1.

**Table 1:** Participant demographics.

Demographics	Total (n = 1938)	UK (n = 986)	Canada (n = 952)	Optimistic Ageing cluster (n = 1035)	Pessimistic Ageing cluster (n = 903)
Age (Mean ± SD)	72.11 ± 5.34	71.32 ± 5.11	72.93 ± 5.45	72.01 ± 5.27	72.22 ± 5.43
Gender (Male)	879	434	445	433	446
Gender (Female)	1056	551	505	602	454
Gender (Other)	3	1	2	0	3
Disability, Yes N, (%)	335 (0.17)	202 (0.1)	133 (0.07)	126 (0.07)	209 (0.11)
Long-term health condition, Yes, N (%)	942 (0.49)	485 (0.25)	457 (0.24)	426 (0.22)	516 (0.27)
Ethnicity (White)	1809	959	850	956	853
Ethnicity (Asian)	78	12	66	50	28
Ethnicity (Black)	14	6	8	7	7
Ethnicity (Mixed race)	12	5	7	6	6
Ethnicity (Prefer not to say)	10	3	7	6	4
Ethnicity (Other)	8	0	8	6	2
Ethnicity (Indigenous)	5	0	5	2	3
Ethnicity (Middle Eastern)	2	1	1	2	0
IMD1	135	71	64	56	79
IMD2	131	70	61	65	66
IMD3	161	71	90	71	90
IMD4	170	71	99	75	95
IMD5	169	70	99	74	95
IMD6	172	70	102	78	94
IMD7	189	72	117	95	94
IMD8	187	70	117	81	106
IMD9	192	70	122	79	113
IMD10	183	68	115	74	109

*UK = United Kingdon; IMD = Indices of Multiple Deprivation; SD = Standard Deviation

**Longitude and Latitude data did not produce a postcode, therefore IMD was not calculated for all participants

### Personality analysis

**Fig 1:**
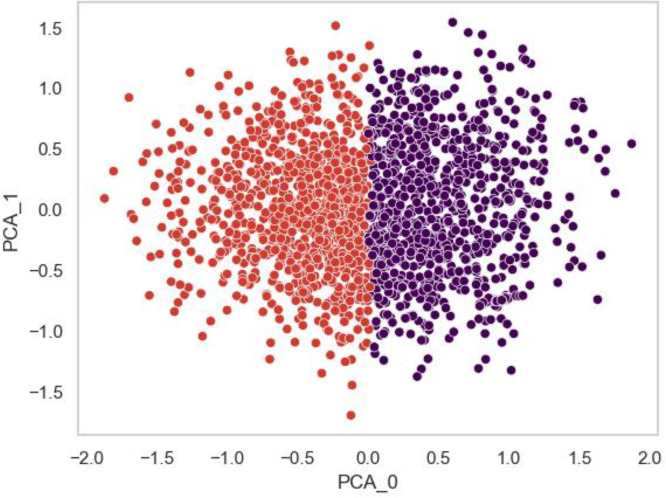
Visualisation of the personality feature clusters of attitudes towards ageing using principal component analysis (PCA). Personality features were used as input to a clustering algorithm (*k*-means) in order to separate participants; red = Optimistic Ageing, purple = Pessimistic Ageing.

**Fig 2:**
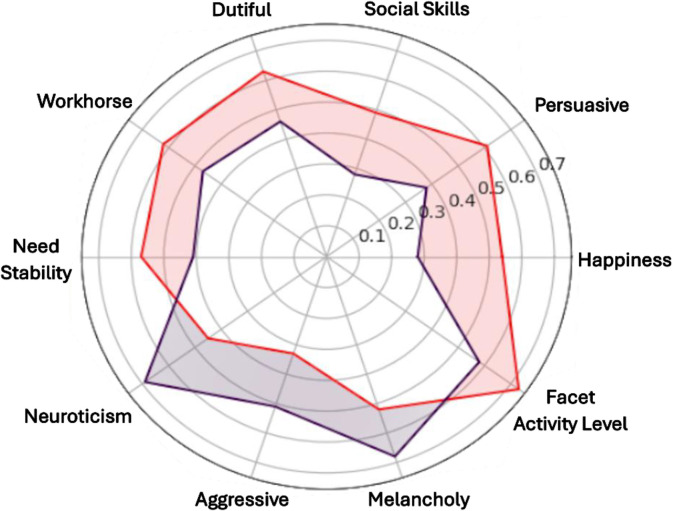
Visualisation of differences in personality attributes between the Optimistic and Pessimistic Ageing personality clusters,; red = Optimistic Ageing, purple = Pessimistic Ageing.

Based on Scaled Insights Behavioural AI analysis, two clusters emerged with distinct personality profiles based on attitudes towards ageing (Fig 1). Cluster 1 (Optimistic Ageing; 53%, n = 552) Canadian and 47%, n = 483) UK respondents) scored higher for happiness, social skills, persuasive, dutiful workhorse, and activity level, compared to Cluster 2 (Pessimistic Ageing; 44%, n = 400) Canadian and 56%, n = 503) UK respondents). Cluster 2 scored higher on neuroticism, melancholy and aggressiveness (see Table 2 for the top ten features and Fig 2 for a visual representation of the different loading of the personality attributes for each cluster; see S1 Table for all 113 personality features). Independent t-tests demonstrate significant differences between the two clusters for the top 10 differentiating personality features (*p* < .001; see Table 2; for all 113 personality features, see S1 Table). Full confidence intervals and p-values for all 113 features are available in S1 Table. There were also significant differences between the two personality clusters for all three of the independent ageing subscales, where Cluster 1 scored higher for physical change, psychosocial loss and psychosocial gain (*p* < .001; see Table 3). A comparison of personality clusters based on demographic variables identified significant differences for gender (*F*(1,168) = 116.14, *p* < .001, *95% CI* [0.62, 0.90], Cohen’s D = −0.15), disability (*F*(1,168) = 8.480, p < .001, *95% CI* [0.38, 0.63], Cohen’s D = −0.27), and long-term health condition (*F*(1,1682) = 48.98, *p* < .001, *95% CI* [0.45, 0.66], Cohen’s D = −0.30). The Optimistic Ageing cluster had a higher proportion of females, and a lower prevalence of disability and long-term health conditions compared to the Pessimistic Ageing cluster. Main effects were also identified for ethnicity (*F*(1,168) = 5.16, *p* < .05, *95% CI* [3.2, 7.12], Cohen’s D = −0.10) and IMD (*F*(1,168) = 50.95, *p* < .001, *95% CI* [48.99, 52.91], Cohen’s D = −0.01), however, pairwise comparisons revealed no significant differences between ethnicity and IMD groups (*p* > .05). No significant differences were found between the personality clusters based on participant age (*p* > .05).

**Table 2:** Cluster centroids for the ten features with greatest absolute value differences between the two personality clusters (mean and standard deviation).

Feature	Optimistic Ageing cluster	Pessimistic Ageing cluster	P Value	Interval for 95% CI	Cohen’s D
Happiness	0.54 (0.19)	0.28 (0.14)	2.88E-199	(-0.27, -0.24)	1.523
Persuasive	0.61 (0.13)	0.38 (0.15)	1.83E-197	(-0.23, -0.21)	1.573
Neuroticism	0.45 (0.27)	0.69 (0.20)	3.33E-94	(0.21, 0.25)	-0.975
Social Skills	0.49 (0.21)	0.28 (0.16)	2.61E-121	(-0.23, -0.19)	1.127
Dutiful	0.63 (0.14)	0.46 (0.12)	1.30E-155	(-0.18, -0.16)	1.135
Workhorse	0.62 (0.18)	0.47 (0.17)	3.91E-71	(-0.17, -0.13)	0.842
Need Stability	0.57 (0.19)	0.41 (0.16)	3.51E-81	(-0.17, -0.14)	0.904
Melancholy	0.52 (0.18)	0.68 (0.15)	2.77E-91	(0.14, 0.17)	-0.963
Aggressive	0.33 (0.13)	0.51 (0.12)	1.76E-183	(0.17, 0.19)	-1.464
Facet activity level	0.73 (0.13)	0.58 (0.14)	6.87E-116	(-0.16, -0.14)	1.125

**Table 3:** Cluster centroids for the three subscales of the AAQ-SF (Laidlaw et al., 2018) between the two personality clusters (mean and standard deviation).

Feature	Optimistic Ageing cluster	Pessimistic Ageing cluster	P Value	Interval for 95% CI	Cohen’s D
Subscale 1: Physical Change	15.04 (3.36)	12.80 (3.84)	<0.001	(-2.56, -1.92)	0.604
Subscale 2: Psychosocial Loss	14.40 (3.52)	12.80 (3.68)	<0.001	(-1.76, -1.12)	0.414
Subscale 3: Psychosocial Gain	17.12 (2.56)	16.00 (2.88)	<0.001	(-1.44, -0.80)	0.412

### Thematic analysis

Participant responses to the five open-ended questions were analysed, with six main themes identified with key differences identified between participants within the Optimistic and Pessimistic personality clusters: (1) attitudes towards physical activity and health, (2) mental health and emotional health, (3) social connections and relationships, (4) independence and autonomy, (5) attitudes towards ageing and mortality and (6) experiences of Ageism. Within the six themes were interconnecting topics and experiences associated with participants perceptions of ageing.

Overall, the Optimistic Ageing cluster were more optimistic and proactive about ageing, focusing on maintaining physical activity, social connections, and independence despite acknowledging physical limitations. They appear more resilient, emphasizing enjoyment of life and positive mental health through proactive coping mechanisms. For instance, many responses from older adults in the Optimistic Ageing cluster reflect a positive outlook on life and ageing, acknowledging that while physical limitations might exist, mental health and maintaining a good attitude are key. Phrases such as like “*I look on every day as anew*” and “*I know my aching bits tell me I’m not but I don’t always listen to it*” reflect optimism despite the challenges that come with ageing. Others highlight the importance of focusing on the good things in life, appreciating the fact that they have lived long and are still able to enjoy their daily routines: “*I consider very lucky to be alive and cancer-free*”.

On the other hand, the Ageing-Pessimist cluster expressed more challenges and concerns related to ageing, particularly around physical decline, chronic illness, and the loss of independence. Mental health struggles, isolation, and reflections on mortality are more prominent in this group, showing a stronger sense of loss compared to the Optimistic Ageing cluster who focus on adaptation and positivity.

For instance, many responses from older adults in the Ageing Pessimistic cluster reflect on how their preferences have changed with age, such as clothing choices and hobbies. For example, one person notes their shift from dresses to more “*comfortable attire*”, while another mentions “*not enjoying activities like concerts or plays anymore*”. Others reported on physical decline and adaptation as they age and needing to accept physical limitations, acknowledging that activities they once enjoyed, like “*sports or long walks, have become more difficult*”. However, many also emphasize adapting to these changes by modifying their routines or finding new ways to stay active: “*I know I need to stay active to maintain quality of life*”.

## Theme 1: Attitudes toward physical activity and health

Participants in both the Optimistic and Pessimistic Ageing personality clusters understand the importance of physical activity, however, the Optimistic Ageing cluster generally takes a more active approach, with individuals continuing to push themselves to stay physically engaged. The Pessimistic Ageing cluster tended to focus more on the limitations that health issues impose, discussing the mental toll of chronic pain and illnesses in greater depth.

### Optimistic ageing (Cluster 1)

Participants in this cluster place significant emphasis on staying physically active as a key to maintaining both physical and mental health. Many participants express their commitment to activities such as walking, gardening, and swimming, despite some acknowledging physical limitations. The approach is proactive, with a focus on keeping the body moving to preserve quality of life: “*I know I need to keep active to maintain quality of life as I age*”. Example quotes are provided below.

“*Without much doubt one’s physical activity promotes both good physical and mental health which in my mind are linked to a significant degree. Being in good physical shape makes you feel better about yourself and hence your mental wellbeing is much better as one looks better, keeping you young at heart!”*

“*Physical activity is definitely a factor in maintain good physical and mental health. It is probably the most important factor in extending our lives and helps to keep our minds sharp. it provides a way to avoid becoming a couch potato and can be an excellent way of meeting people. it also helps build self-confidence”.*

Optimistic Ageing participants also acknowledged the challenges of ageing, there’s a greater sense of acceptance in slowing down but adapting to it. They understand their limitations but continue with modified routines, demonstrating resilience. Example quotes are provided below.

“*Slowing down in my older age is somewhat of a treat for me. Have been a workaholic my entire life. The further into my 6th decade of life, I am experiencing a peace increasingly taking over which is causing me to relax, understand it is ok to just do nothing”*

“*Even in my 50s, I accept and recognise I am slowing down, but once I get going I am still able to walk quickly and for several miles. But I accept that it is not what it was but that is no reason to stop. Recognise your strengths and play to them”*

### Pessimistic ageing (Cluster 2)

While there is an emphasis on staying active, participants in the Pessimistic Ageing cluster report a greater focus on the challenges posed by physical health issues such as chronic pain, arthritis, or serious medical conditions. They discuss how these limitations impact their ability to stay motivated, but they recognize that staying active is still important: “*I try to walk a bit most days but find myself more limited by pain*”. Example quotes are provided below.

“*My back is stiff and sore due to arthritis and long ago vertebrae fractures. Hips and knees are sore. The usual old age stuff. Demotivation is mostly pain. Bad back, worn out knees and hips, and my inability to raise my hands above my head for more than a few minutes all limit what I am willing and able to do. I am aware moderate exercise is beneficial... [but I] view exercise as a chore to be done in order to prolong the inevitable decline as I age. I have never noticed any mental benefit to exercise”.*

*“Have arthritis so I can’t golf anymore and that was my main activity and I don’t work anymore because I’m too old. I can’t lift heavy things anymore. I used to walk a lot but now with my arthritis I never know how far I can go or if I can go anywhere. Yes, I hurt too much from time to time from arthritis and my blood pressure is way too high. I had a stroke and I’m afraid of having another one. I have to take blood thinners, blood pressure, meds, and cholesterol pills daily. So I went from taking no medications to taking all this crap and I hate it. Some days I feel like I’m just sitting around waiting to die. I used to be able to walk a lot now because of my arthritis that is an impossible task most days*”

*“From being physically active to never going to the gym and exercising. i used to play sport regularly but now I just watch. My physical condition is steadily worsening and I have more hospital appointments than ever. My health prevents me from doing any vigorous exercise. i had bowel cancer which means I use a colostomy bag which limits my exercise. Later I developed a hernia. my physical health has steadily got worse and I have found it hard to be active. my health has deteriated [sic] over years. i suffer from diabetes 2. high blood pressure, high collestral [sic],kidney problems and regular visits to hospital. just recently I was hospitalised with burst ulcers”.*

A key difference in the Pessimistic Ageing Cluster was the discussions of serious illnesses like cancer or other chronic diseases that greatly impact mobility and motivation. Participants discussed physical decline, such as struggling with stairs or needing mobility aids, is more of a central theme. Example quotes are provided below.

*“I tire more readily and lack energy! My lack of mobility is an issue and does not allow me to do some of the things, (i.e.,; travel) I would like to do! I only have 30% hearing in one ear and wear a hearing aid making it difficult to hear conversations causing isolation. I love to travel and go on trips, but airports are daunting, stairs are impossible and energy is lacking. [on physical activity] The lack of energy prevails and I don’t do it! The ambition is there but I am just too tired and sore to do anything! It hurts to move, my back aches and my legs ache and my feet are numb! I am also a bit depressed because of constant pain and it all becomes a vicious circle!”*

*“Things get harder to do such as tieing[sic] shoes, putting on socks, putting on pants. Stairs get harder to climb, shower get harder to step into. Lifting heavy grocery bags or items get more difficult. You read that exercise will help with so many things like anxiety, stress etc. which I feel is a lot of BS. I golf and still get sore muscles and stiff the next day, I have sore knees, hands, back. The more I use them I keep getting sore”.*

## Theme 2: Mental and emotional health

Participants in the Optimistic Ageing cluster have a more optimistic and proactive approach to mental health, tying it to both physical activity and social connections. Participants in the Pessimistic Ageing cluster report significant struggles with mental health, where emotional well-being is more impacted by chronic health conditions, leading to challenges like depression and anxiety.

### Optimistic ageing (Cluster 1)

Within this cluster was a strong and consistent theme of maintaining a positive outlook and mental engagement through activities such as reading, doing puzzles, or socializing with friends. Participants speak about mental health in a positive, solution-oriented way, often tying mental well-being to physical activity. Many are proactive in engaging in hobbies and staying busy to maintain their mental health. For example,

“*I try to stay engaged by reading, doing puzzles and gardening. I try to spend more time with family and friends. As I get older I realize that you have to savour moment and make the best of every opportunity. I feel that to do so you have to make sure that you stay healthy and active. Keep your mind sharp to make sure that you are able to make decision for yourself”.*

Optimistic Ageing participants largely express acceptance of ageing and approach it with a certain level of resilience. They are more likely to mention strategies for coping, such as keeping a positive outlook and enjoying life despite physical limitations. For instance,

“*Nap when I am tired, accept that I can’t get as much done in one day as I used to, don’t be as hard on myself when I don’t accomplish everything I had hoped to, relax more... and be happy. Be sure to laugh and find joy in every day. You need good physical, emotional and psychological health. My husband and I try to get regular exercise because we both feel very strongly that there are many benefits - increased strength, better mental health, better sleep, more resilience*”.

### Pessimistic ageing (Cluster 2)

While some individuals in the Pessimistic Ageing cluster express optimism, mental health issues such as depression and anxiety were more prominent, often linked to their declining physical health. Some participants explicitly mention battling mental health issues due to their physical limitations or chronic illnesses: “*I feel like giving up sometimes because of my health, but I keep trying*”. For instance,

“*I have been told that old people should have a suicide pact to stop the drain on the health care system. I hate myself! I have depression, rarely motivated at all. I used to walk a few miles a day, now I hate the idea of seeing people, especially the new immigrants who are here for 15 minutes and feel they are entitled. I guess I am basically lazy, maybe that’s why I don’t exercise. I feel mostly alone in life, I just don’t care about my health, or my wellbeing. I live with pain, and Docs will not prescribe the meds I used to have that eased the pain. My life sucks”.*

Participants in the Pessimistic Ageing cluster discussed mental engagement and strategies to cope with mental health issues, however, this was much less, and they focused more on coping with mental health challenges than on active participation in mentally stimulating activities. For example,

“*I can’t do as much as I would like to do. I don’t dress up anymore or put much make up on. I worry about my health more. I worry more in general. I don’t always feel safe. You need the money to cope and need to be able to keep up with household jobs. I think physical activity is very important but if you are not able to do it then you can’t. Walking is the best I can do and cannot do as much a when I was younger. It does affect both physical and mental health. Sitting in a house all day is very bad for you but some elderly do not have a choice”.*

## Theme 3: Social connections and relationships

The Ageing-optimists value and actively seek social connections as a part of their healthy ageing process, while Cluster 2 shows more signs of isolation or reduced socialization due to either physical decline or caregiving responsibilities. The social life of Cluster 1 appears to be more vibrant and integrated into their coping mechanisms, while Cluster 2 reflects more challenges in maintaining these social bonds.

### Optimistic ageing (Cluster 1)

In the Optimistic Ageing cluster, participants placed high value on social interaction, often mentioning friendships, family, or community activities. Socializing is seen as an important part of life, contributing to both mental and emotional well-being. Volunteering, spending time with friends, and maintaining a supportive network are key parts of their lives. Example quotes are provided below.

“*Enjoy volunteering in my community as it makes me feel I am still contributing and helping others. Having a number of good friends and neighbours to do activities with gives support in my life. Try to get out most days to exercise or meet with others so I won’t just sit in my home”.*

*“Cooking, cleaning, shopping and lots of friends to experience outings with for a good social life. Having good support from my family should I need help. Having a good health provider that can recognize issues before they become catastrophic. Good community supports for seniors”.*

Many participants in this cluster seem to strike a balance between maintaining independence and staying socially engaged. They express a desire for companionship but are also comfortable being alone and enjoying solitude when necessary. For instance,

“*It is important to remain as independent as possible, to work to the best of your ability in body and mind. It is important to manage your health and finances, to keep your pantry well stocked, your closets full but not to over flowing with appropriate wear for all seasons. it is important to challenge your mind everyday, to try new adventures, to cook those recipes that would otherwise take too long when raising a family”.*

### Pessimistic ageing (Cluster 2)

In contrast to the Optimistic Ageing cluster, solitude or reduced socialization is a more prominent theme in the Pessimistic Ageing cluster. Some individuals seem to struggle with loneliness, with a few enjoying their time alone but others lamenting the loss of social connections or feeling isolated due to physical decline: “*I feel alone sometimes and miss having more company*”. For example,

“*I do worry that as I get much older my health and mobility could fail and I could developed other health issues not in my control. I worry about loneliness and isolation. I find if the weather is really bad on a particular day and I can’t go out I feel listless, bored and quite isolated”.*

Participants in this cluster also reported a greater emphasis on family caregiving compared to the Optimistic Ageing cluster, where individuals are either receiving care or providing care to loved ones, which sometimes limits their ability to socialize outside the home. Example quotes are provided below.

*“My role at home has become that of a caregiver and as such I haven’t been giving thoughts to such a thing [socializing by volunteering for a ESL class for newcomers]. My not engaging in physical activities is not attributed to my physical or mental state but to the demands on my time”.*

*“I live alone now and really enjoying the calm. My purpose these days are as a caregiver for my 95 year old father and my 93 year old Aunt. My mother passed away in 2014 after suffering an inoperable cancer for 5 years. I will continue to live alone until things change”.*

## Theme 4: Independence and autonomy

Whilst both personality clusters emphasize the importance of maintaining independence, participants in the Optimistic Ageing cluster tend to approach it with a more proactive and optimistic attitude, whereas the participants in the Pessimistic Ageing cluster exhibit more concerns and difficulties regarding the loss of autonomy. Participants in the Pessimistic Ageing cluster are also more likely to have already experienced significant declines in independence and express the emotional toll this takes.

### Optimistic ageing (Cluster 1)

Independence is a major theme for participants in the Optimistic Ageing cluster, with many individuals discussing strategies for remaining self-sufficient for as long as possible. Some have already made adjustments (downsizing homes, using mobility aids), but they emphasize the importance of living independently: “*I want to live in my own home as long as I can*”. Example quotes are provided below.

“*I believe I have prolonged my agility by keeping my joints mobile and my mental state positive through moving a lot everyday. I want to live in my own home as long as possible and to be independent. We truly believe in the saying if you don’t use it you lose it so we push ourselves to be active and it adds to our positivity”.*

*“I want to live in my own home for as long as I can, so keeping myself fit and healthy is important”.*

Despite physical limitations, many participants express optimism about being able to maintain their autonomy and make the necessary adjustments to do so. For instance,

“*Worsening health conditions have an impact as I live with chronic pain... but a determined attitude to carry on sees me through. I accept the declining condition despite battling it as it is all part of the human condition and an inevitable consequence of living so long. I am motivated by role models and a desire to never give in”.*

### Pessimistic ageing (Cluster 2)

For participants in this cluster, losing independence seems more prominent, especially for those dealing with serious health issues. Some express fear or anxiety about losing their independence and having to rely on others, while others already need assistance and express feelings of frustration or sadness about it. For example,

*“I feel scared when I see people in wheelchairs or rely on others to feed them. I pay more attention now when I see people in seniors’ homes”.*

Many participants in the Pessimistic Ageing cluster report having to make practical adjustments in relation to ageing, such as moving to a different type of home (e.g., bungalows, assisted living) or relying on family and caregivers to help with daily tasks. For example,

*“I have moved into my “forever” home, a bungalow, in the last 7 months, making preparations for any decline in my mobility that might come as I get older, so that I can retain my independence and not become a burden on my family”.*

## Theme 5: Attitudes toward ageing and mortality

The Ageing-optimists generally exhibit a more positive and resilient attitude toward ageing, focusing on continuing to enjoy life. The Ageing-optimists report greater anxiety and concern about mortality, with a stronger sense of loss and limitation as they face more severe health challenges and declining independence.

### Optimistic ageing (Cluster 1)

Participants in the Optimistic Ageing cluster generally approach ageing with a sense of optimism and acceptance. They emphasize enjoying life, focusing on hobbies, friendships, and self-care. The theme of ageing gracefully is common, with many embracing their current stage of life without too much fear of mortality: “*I don’t feel old yet, even though I know I am*”. For example,

“*I live by the motto that aging is mandatory but maturity is optional. I refuse to grow up. Aging should not mean stopping what you enjoy doing. You may need to slow down a bit, depending on your physical condition. Don’t be afraid to ask for help if and when you need it. No I have not changed my view of myself. I don’t feel old”.*

*“I have become more confident as I have got older but I don’t feel older and sometimes I forget that I am aging gracefully”.*

There was also a consistent theme of resilience against physical decline, with participants finding ways to enjoy their lives despite limitations. Ageing is seen as a natural process, but one that can still include joy and fulfilment. Example quotes are provided below.

*“Even though my lower back is in constant pain and both my hips are damaged, I know that walking is good for those conditions, so I keep trying... I get a sense of accomplishment when I achieve a physical goal, like walking for ten minutes before breakfast every day”.*

*“I am always motivated towards any form of physical activity for the sheer pleasure of it and always push myself to my own personal limit which is limited to an extent because overexertion causes pain. In spite of the pain I feel its extremely important to keep moving and not to give up”.*

“*A positive attitude is essential for successful aging. Naturally we all experience aches, pains and various ailments but it’s how we deal with them that counts. Keeping a social network (friends, family) is important. They give us love and support so that we know that we’re not alone. When I look in the mirror I see the wrinkles but I don’t feel like an old person. I never have negative thoughts about myself; I feel like I’m much younger than my actual age”.*

### Pessimistic ageing (Cluster 2)

More Reflection on Mortality: Mortality is a more present concern in Cluster 2, with individuals expressing worries about their declining abilities, serious health conditions, and the impact of ageing on their quality of life. Some explicitly mention the fear of death or the anxiety that comes with seeing their health decline: “*I feel more aware of how close the end is now*”. For instance,

“*I worry about how my health will change in the future and if I will be able to cope I’m scared. I’m worried how it will affect my family in my changing of capabilities. I’m scared of old age illnesses”.*

Pessimistic Ageing participants also discussed loss more compared to those in the Optimistic Ageing cluster, whether it’s the loss of physical ability, independence, or even the loss of loved ones. These reflections on mortality often tie back to a sense of limitation and frustration with ageing. For example,

“*I am a widow if I could change that and have my husband back I would love that. I feel very lost without him. Same with sleep has been an issue with me since I lost my husband as I witnessed his death but I don’t have much longer on this world so why shouldn’t I be able to sleep well?”.*

*“Since becoming a widow in 2017, I have been far more careful to avoid confrontation of any kind. I’ve seen so many friends’ health begin to fail and before you know it they are being moved to a residential or nursing home, where they usually pass away within a couple of years”.*

*“As I have gotten older my life took a new direction that I didn’t expect due to the passing of my wife, who was my true love and best friend. We had many plans for retirement and now that she is no longer here, those plans have not developed as expected. I think if my wife was still alive, I would be more active as we would be doing things together”.*

## Theme 6: Experiences of ageism

The final theme identified was experiences of ageism which was reported by both personality clusters. Older adults in both clusters reported that ageism was a challenge that impacted several areas of their lives including their ability to maintain independence, engagement in physical activity and participation in the workplace. As this was consistent across both clusters, quotes are provided from both.

“*I feel that there is a lot of ageism in the world now, even the government disrespects pensioners by not paying a better pension and threatening to make us take another driving test and theory test in 2027*”. (Optimistic Ageing cluster)

“*I have also experienced age discrimination which was startling to me... I still feel ultimately like the same person and equally like I am still learning in life and enjoy that growth*”. (Optimistic Ageing cluster)

“*It is ageism for people to write off older folks who are not as agile as younger people and then consider them physically unfit for failure to keep up with the standards they set as youth”*. (Pessimistic Ageing cluster)

*“I love participating in physical activity for my own wellbeing and to prove to others that my age is not a barrier when undertaking physical activities. What demotivates me is when people automatically assume I will not be able to join in. There is too much ageism at the minute*”. (Pessimistic Ageing cluster)

“*I wanted to stay in the work force for much longer and as my companies were acquired and I was laid off, it became increasingly more difficult to find employment as ageism is alive and well... my experience was no longer appreciated, and work was given to the lowest bidder”*. (Pessimistic Ageing cluster)

## Discussion

This study examined whether personality is a predictor of attitudes towards ageing in a sample of adults aged 65 years and older from the UK and Canada. Study findings demonstrate that first the Behavioural AI analysis is capable of assessing personality attributes associated with perceptions of attitudes towards ageing, and second that there is a significant difference in the attitudes of UK and Canadian adults aged 65 years and over in their perceptions of physical change, psychosocial loss and psychosocial gain as they age.

This study has used an innovative Behavioural AI solution to examine the association between personality and attitudes towards ageing, that both supports previous research highlighting that personality is a factor that influences these attitudes and may consequently increase risks and behavioural responses associated with ageing (e.g., reduced social engagement, loneliness, reduced physical activity) but also extends previous research by clustering personality to examine the combined effect of personality attributes rather than singularly exploring the relationship. The Scaled Insights Behavioural AI analysis, which considers personality traits alone, highlights significant differences in perceptions of ageing, i.e., perceptions of physical change, psychosocial loss and psychosocial gain. The use of Scaled Insights Behavioural AI solution also highlights the importance and use of language to understand the personalities of older adults that influences attitudes towards ageing; an approach that to our knowledge has not been employed previously.

1There is a need to consider personality in the approaches used to supporting older people in the Pessimistic Ageing personality cluster to address the significantly more negative attitudes towards ageing across the three subscales of the AAQ-SF [18] compared to the Optimistic Ageing personality cluster; physical change, psychosocial loss and psychosocial gain. Previous research examining perceptions of ageing have identified optimism as an important construct that can promote healthy, active ageing whilst buffering against physical and mental health decline associated with ageing [26]. For instance, a Wurm and Benyamini conducted a survey of perceptions of ageing in a representative population sample of German adults aged 40–85 years reporting that optimism was an important construct in reducing the impact of ageing on health, where optimists were better able to maintain physical functioning and lower depressive symptoms [27].

Several themes that emerged in the open-ended responses highlight the impact and emotions associated with negative attitudes towards ageing, and how this impacts older adults’ societal engagement and identity, which was particularly evident for the Pessimistic Ageing cluster. Older adults within the Pessimistic Ageing cluster reported more issues related to ageing reflecting the more negative attitudes towards ageing compared to the Optimistic Ageing cluster as measured in the AAQ-SF [18], as well as the consequences including the greater restriction and behavioural changes they needed to make to adjust to ageing. They also discussed coping strategies less when describing similar themes and issues relating to ageing as the participants in the Optimistic Ageing cluster. Both personality clusters discussed the issue of ageism, with specific examples that reflect a perception and, in some instances, experiences of ageism from others that impacts their participation in society.

In line with previous research [5,6,10] our study findings highlight the importance of individuals attitudes towards their ageing and in doing so, we call for efforts to promote positive perceptions of ageing and intervention that address barriers that may exacerbate or increase the risk of negative perceptions and behaviours associated with ageing.. Interventions that specifically target and communicate effectively with older adults are needed, that are designed based on personality profiles of older adults as highlighted in this study, may prove to be more effective in promoting healthy, active ageing. Addressing ageism and age-related stereotypes appears warranted given that older adults in both personality clusters reported these negative experiences. A systematic review of studies examining the effects of age-related stereotypes and health outcomes of adults aged 50 years and above, highlighted that interventions can be effective in improving self-perceptions of ageing, physical function and physical activity adoption [27,28]. Interventions may also focus on self-help both as a preventative intervention and as a support tool for older adults to provide tailored support for people experiencing issues associated with ageing. These would include the acceptance of and strategies to deal with physical decline or retirement from work. Interventions are also needed that support the increased risk of mental health concerns, where for instance cognitive behavioural therapy could be tailored and potentially personalised to older adults to support them to manage these challenges and the associated emotions. Those interventions may initiate new online communities that are psychologically informed, inviting participation and offering group work that builds relationships and facilitates in-person meetings and events. This current study has several strengths, in particular the use of a novel Behavioural AI solution to assess personality attributes and in doing so, provide a unique assessment of the potential role of personality on attitudes towards ageing. Given the importance of attitudes towards ageing on the health and wellbeing of individuals as well as communities, there are implications of these findings for policymakers and organizations working to promote healthy ageing including the tailoring of efforts to communicate and support people as they age more effectively. Future research and applied efforts should explore how to develop these tailored approaches that improve the perceptions of ageing, specifically perceived physical change, psychosocial loss and psychosocial gain, given the importance of these perceptions for attitudes towards ageing.

This study is not without its limitations. First, participants were recruited using an online participant pooling mechanism which may mean that the sample recruited doesn’t include individuals who experience digital deprivation. Whilst this may be the case, IMD data suggests that people from across the spectrum of deprivation have been recruited including individuals within the 10–20% most deprived communities. Second, the study examined attitudes towards ageing rather than objective impacts of ageing and thus, these perceptions may not translate into poorer physical change, psychosocial loss and lower psychosocial gain reported by older adults in the Pessimistic-Ageing personality cluster. However, previous research has highlighted the importance and the clear relationship between perceptions of ageing with poorer health, functioning and behavioural outcomes and thus, addressing more negative attitudes towards ageing is warranted. Third, the study employed self-report measures and thus, there is a potential that social desirability may have influenced participants responses. Fourth, there were more participants in the Pessimistic Ageing cluster who reported having a disability and long-term health condition, and thus the more Pessimistic Ageing personality attributes identified in this cluster may be associated. There are also more participants who reported a disability in the UK (10%) compared to the Canadian (7%) sample, which may reflect the higher number of UK participants in the Pessimistic Ageing compared to Optimistic Ageing cluster. However, whilst there were more people in the Pessimistic Ageing cluster with a disability, the majority of participants in both personality clusters reported no disability. Furthermore, the qualitative analysis highlighted that for many participants without a disability, differences in their perceptions of ageing including concerns and barriers to healthy behaviour adoption were apparent. Another limitation is the lack of ethnic diversity in the dataset, as most participants identify as white. Given that cultural and ethnic backgrounds can influence attitudes toward ageing, the findings may not fully capture the perspectives of more diverse populations. Additionally, as the study employed a cross-sectional design, we are unable to provide causal inferences between personality and attitudes towards aging. Finally, the Behavioral AI used to derive personality traits is based on statistical patterns learned from language data. As with many machine learning systems, the models’ outputs reflect the structure of the training data, meaning that some demographic groups or linguistic styles might be overrepresented.

In summary, this study has utilised an innovative Behavioural AI solution to examine whether personality attributes are related to attitudes towards ageing. The study demonstrates that personality is related to perceived physical change, psychosocial loss and psychosocial gain and that based on personality there are significant differences in attitudes towards ageing in older adults aged 65 years and over. There are key implications of the findings presented including the consideration of personality in the impact of ageing as well as interventions to promote and support healthy ageing, and there is a need to address ageism that is a key issue impacting older adults’ participation in many spheres of society.

## Supporting information

S1 Fig Percentage of participants based on level of deprivation in Canada and the UK. (JPG)

S2 Fig Indices of multiple deprivation by the two personality clusters. (JPG)

S1 Table 113 personality scores by cluster, with p-value, CI interval. (DOCX)
