# Supplementary figures and images for: Exploring the association between personality and attitudes towards ageing in UK and Canadian older adults’: Use of a novel Behavioural Artificial Intelligence solution
Source: PLoS One. 2026 Apr 29;21(4):e0347422. doi: 10.1371/journal.pone.0347422 (PMC13127943; doi:10.1371/journal.pone.0347422)

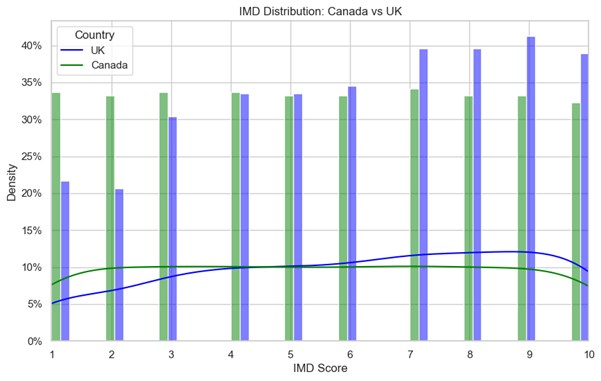

Supplement: S1 Fig — (JPG) [file pone.0347422.s001.jpg]

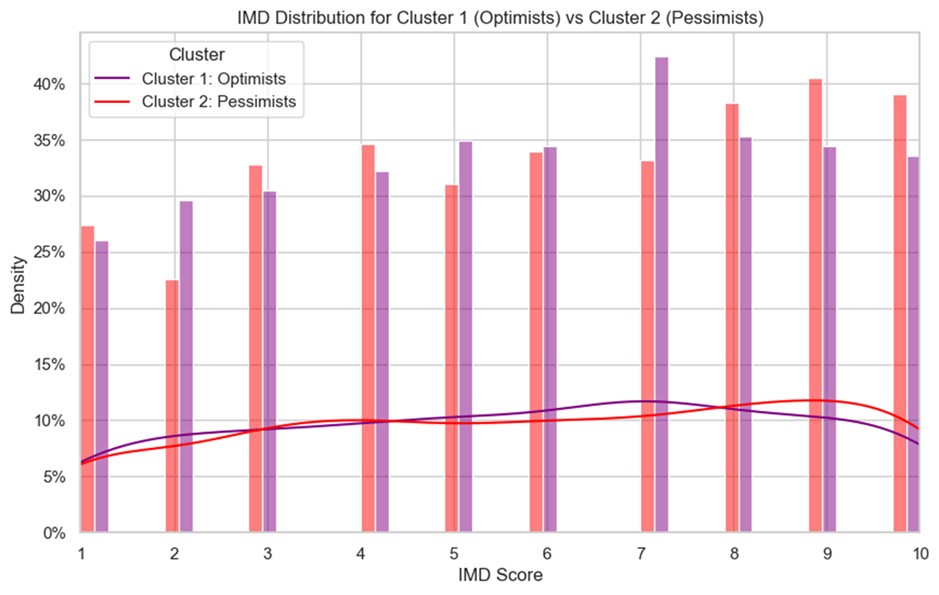

Supplement: S2 Fig — (JPG) [file pone.0347422.s002.jpg]
