# Supplementary material for: Exploring the association between personality and attitudes towards ageing in UK and Canadian older adults’: Use of a novel Behavioural Artificial Intelligence solution
Source: PLoS One. 2026 Apr 29;21(4):e0347422. doi: 10.1371/journal.pone.0347422 (PMC13127943; doi:10.1371/journal.pone.0347422)
Supplement: S1 Table — (DOCX) [file pone.0347422.s003.docx]

**S1 Table:** 113 personality scores by cluster, with p-value, CI interval

| **Personality Traits** | **Optimistic Ageing cluster** | **Pessimistic Ageing cluster** | **P Value** | **Interval for 95% CI** | **Cohen’s D** |
| --- | --- | --- | --- | --- | --- |
| Happiness | 0.54 (0.19) | 0.28 (0.14) | 2.88E-199 | (-0.27, -0.24) | 1.523 |
| Persuasive | 0.61 (0.13) | 0.38 (0.15) | 1.83E-197 | (-0.23, -0.21) | 1.573 |
| Neuroticism | 0.45 (0.27) | 0.69 (0.20) | 3.33E-94 | (0.21, 0.25) | -0.975 |
| Social Skills | 0.49 (0.21) | 0.28 (0.16) | 2.61E-121 | (-0.23, -0.19) | 1.127 |
| Dutiful | 0.63 (0.14) | 0.46 (0.12) | 1.31E-155 | (-0.18, -0.16) | 1.135 |
| Workhorse | 0.62 (0.18) | 0.47 (0.17) | 3.91E-71 | (-0.17, -0.13) | 0.842 |
| Need Stability | 0.57 (0.19) | 0.41 (0.16) | 3.51E-81 | (-0.17, -0.14) | 0.904 |
| Melancholy | 0.52 (0.18) | 0.68 (0.15) | 2.77E-91 | (0.14, 0.17) | -0.963 |
| Aggressive | 0.33 (0.13) | 0.51 (0.12) | 1.76E-183 | (0.17, 0.19) | -1.464 |
| Facet activity level | 0.73 (0.13) | 0.58 (0.14) | 6.87E-116 | (-0.16, -0.14) | 1.125 |
| Cold | 0.36 (0.14) | 0.51 (0.15) | 2.13E-103 | (0.14, 0.17) | -1.053 |
| Facet achievement striving | 0.71 (0.11) | 0.56 (0.12) | 2.30E-139 | (-0.16, -0.14) | 1.266 |
| Depression | 0.54 (0.14) | 0.69 (0.16) | 1.19E-90 | (0.13, 0.16) | -0.985 |
| Facet self-efficacy | 0.55 (0.18) | 0.41 (0.18) | 2.55E-57 | (-0.15, -0.12) | 0.752 |
| Conscientiousness | 0.80 (0.10) | 0.66 (0.12) | 2.55E-133 | (-0.15, -0.13) | 1.235 |
| Insecure | 0.38 (0.14) | 0.52 (0.13) | 6.07E-109 | (0.13, 0.16) | -1.074 |
| Facet trust | 0.53 (0.10) | 0.40 (0.10) | 1.59E-149 | (-0.14, -0.12) | 1.299 |
| Active | 0.44 (0.12) | 0.31 (0.11) | 1.73E-110 | (-0.14, -0.12) | 1.075 |
| Need harmony | 0.44 (0.19) | 0.31 (0.15) | 2.19E-59 | (-0.14, -0.11) | 0.756 |
| Extraversion | 0.35 (0.12) | 0.22 (0.09) | 6.39E-136 | (-0.14, -0.12) | 1.209 |
| Trusting | 0.41 (0.12) | 0.28 (0.09) | 3.64E-143 | (-0.14, -0.12) | 1.247 |
| Reward bias | 0.54 (0.14) | 0.42 (0.14) | 1.27E-75 | (-0.13, -0.11) | 0.873 |
| Facet cheerfulness | 0.20 (0.12) | 0.08 (0.06) | 2.75E-148 | (-0.13, -0.11) | 1.267 |
| Facet self-discipline | 0.26 (0.13) | 0.14 (0.09) | 1.59E-111 | (-0.13, -0.11) | 1.070 |
| Facet assertiveness | 0.61 (0.14) | 0.49 (0.16) | 6.83E-64 | (-0.14, -0.11) | 0.809 |
| Sociable | 0.46 (0.12) | 0.35 (0.11) | 8.33E-80 | (-0.11, -0.09) | 0.898 |
| Big5 conscientiousness | 0.26 (0.13) | 0.15 (0.11) | 1.33E-88 | (-0.13, -0.10) | 0.944 |
| Facet anger | 0.21 (0.10) | 0.32 (0.14) | 7.26E-72 | (0.09, 0.11) | -0.878 |
| Organized | 0.68 (0.10) | 0.58 (0.10) | 9.50E-89 | (-0.11, -0.09) | 0.958 |
| Cheerful | 0.51 (0.09) | 0.41 (0.08) | 8.61E-125 | (-0.11, -0.09) | 1.167 |
| Adventurous | 0.61 (0.08) | 0.51 (0.08) | 9.54E-140 | (-0.11, -0.09) | 1.258 |
| Cooperative | 0.75 (0.09) | 0.65 (0.11) | 3.44E-105 | (-0.12, -0.10) | 1.070 |
| Ambitious | 0.78 (0.10) | 0.68 (0.11) | 2.39E-90 | (-0.11, -0.09) | 0.981 |
| Facet altruism | 0.82 (0.10) | 0.72 (0.12) | 1.01E-84 | (-0.11, -0.09) | 0.957 |
| Need structure | 0.66 (0.14) | 0.56 (0.15) | 2.78E-48 | (-0.11, -0.09) | 0.690 |
| Power driven | 0.58 (0.21) | 0.48 (0.20) | 1.20E-25 | (-0.12, -0.08) | 0.483 |
| Type A | 0.34 (0.32) | 0.44 (0.33) | 1.01E-12 | (0.08, 0.14) | -0.328 |
| Facet depression | 0.84 (0.10) | 0.93 (0.06) | 2.75E-120 | (0.09, 0.10) | -1.115 |
| Facet anxiety | 0.70 (0.14) | 0.79 (0.12) | 2.67E-48 | (0.08, 0.10) | -0.678 |
| Energetic | 0.39 (0.12) | 0.30 (0.12) | 1.15E-53 | (-0.10, -0.08) | 0.727 |
| Facet adventurousness | 0.50 (0.15) | 0.41 (0.16) | 3.58E-33 | (-0.10, -0.07) | 0.559 |
| Need self-expression | 0.45 (0.19) | 0.36 (0.17) | 1.49E-31 | (-0.11, -0.08) | 0.538 |
| Need challenge | 0.40 (0.19) | 0.31 (0.16) | 4.61E-31 | (-0.11, -0.08) | 0.532 |
| Need love | 0.54 (0.22) | 0.45 (0.20) | 7.03E-19 | (-0.10, -0.07) | 0.406 |
| Need liberty | 0.37 (0.15) | 0.28 (0.13) | 2.77E-45 | (-0.10, -0.08) | 0.653 |
| Value openness to change | 0.58 (0.18) | 0.49 (0.18) | 1.76E-32 | (-0.11, -0.08) | 0.550 |
| Leisure oriented | 0.77 (0.14) | 0.69 (0.15) | 1.93E-29 | (-0.09, -0.06) | 0.527 |
| Value conservation | 0.22 (0.14) | 0.14 (0.10) | 2.29E-51 | (-0.10, -0.07) | 0.693 |
| Family oriented | 0.57 (0.17) | 0.49 (0.19) | 2.71E-20 | (-0.09, -0.06) | 0.428 |
| Facet cooperation | 0.64 (0.12) | 0.57 (0.13) | 4.41E-35 | (-0.08, -0.06) | 0.577 |
| Emotionally aware | 0.58 (0.14) | 0.65 (0.13) | 2.05E-33 | (0.06, 0.09) | -0.556 |
| Facet friendliness | 0.11 (0.09) | 0.04 (0.05) | 1.92E-98 | (-0.08, -0.07) | 0.990 |
| Friendly | 0.51 (0.09) | 0.44 (0.09) | 5.34E-55 | (-0.07, -0.06) | 0.738 |
| Facet self-consciousness | 0.87 (0.10) | 0.94 (0.06) | 7.25E-76 | (0.06, 0.08) | -0.856 |
| Stressed | 0.64 (0.11) | 0.71 (0.11) | 2.81E-46 | (0.06, 0.08) | -0.667 |
| Friend focus | 0.57 (0.11) | 0.50 (0.11) | 5.31E-43 | (-0.08, -0.06) | 0.645 |
| Facet immoderation | 0.64 (0.15) | 0.71 (0.14) | 1.54E-25 | (0.06, 0.08) | -0.480 |
| Facet vulnerability | 0.82 (0.11) | 0.88 (0.09) | 1.05E-38 | (0.05, 0.07) | -0.598 |
| Facet dutifulness | 0.67 (0.12) | 0.61 (0.14) | 2.68E-18 | (-0.06, -0.04) | 0.406 |
| Big5 extraversion | 0.11 (0.09) | 0.05 (0.06) | 1.84E-54 | (-0.06, -0.05) | 0.711 |
| Big5 neuroticism | 0.20 (0.12) | 0.14 (0.11) | 1.04E-26 | (-0.07, -0.05) | 0.492 |
| Need closeness | 0.42 (0.16) | 0.36 (0.14) | 2.31E-19 | (-0.08, -0.05) | 0.412 |
| Facet excitement seeking | 0.43 (0.16) | 0.37 (0.15) | 2.10E-13 | (-0.07, -0.04) | 0.335 |
| Sexual focus | 0.54 (0.17) | 0.60 (0.16) | 5.71E-16 | (0.05, 0.08) | -0.370 |
| Independent | 0.55 (0.16) | 0.61 (0.16) | 2.86E-15 | (0.04, 0.07) | -0.362 |
| Facet artistic interests | 0.78 (0.13) | 0.73 (0.15) | 1.34E-15 | (-0.06, -0.04) | 0.371 |
| Facet morality | 0.74 (0.17) | 0.69 (0.19) | 6.12E-12 | (-0.07, -0.04) | 0.318 |
| Big5 agreeableness | 0.23 (0.15) | 0.18 (0.12) | 2.89E-18 | (-0.07, -0.04) | 0.395 |
| Genuine | 0.38 (0.09) | 0.33 (0.11) | 7.89E-29 | (-0.06, -0.04) | 0.522 |
| Assertive | 0.30 (0.12) | 0.35 (0.14) | 5.40E-15 | (0.04, 0.06) | -0.364 |
| Value self-transcendence | 0.82 (0.10) | 0.77 (0.13) | 1.44E-22 | (-0.06, -0.04) | 0.459 |
| Facet emotionality | 0.82 (0.12) | 0.77 (0.13) | 8.32E-15 | (-0.06, -0.03) | 0.359 |
| Cautious | 0.76 (0.08) | 0.80 (0.09) | 2.82E-26 | (0.04, 0.05) | -0.493 |
| Intellectual | 0.48 (0.08) | 0.52 (0.10) | 4.50E-20 | (0.03, 0.05) | -0.428 |
| Disciplined | 0.56 (0.10) | 0.52 (0.10) | 1.44E-16 | (-0.05, -0.03) | 0.381 |
| Facet sympathy | 0.87 (0.10) | 0.83 (0.12) | 1.79E-14 | (-0.05, -0.03) | 0.355 |
| Self-assured | 0.55 (0.14) | 0.51 (0.13) | 1.69E-07 | (-0.04, -0.02) | 0.238 |
| Imaginative | 0.28 (0.07) | 0.32 (0.08) | 3.85E-35 | (0.04, 0.05) | -0.585 |
| Need excitement | 0.37 (0.18) | 0.33 (0.17) | 1.32E-07 | (-0.06, -0.03) | 0.241 |
| Liberal | 0.53 (0.12) | 0.57 (0.11) | 3.11E-16 | (0.03, 0.05) | -0.372 |
| Agreeableness | 0.40 (0.08) | 0.37 (0.09) | 3.89E-15 | (-0.04, -0.02) | 0.362 |
| Work oriented | 0.73 (0.08) | 0.70 (0.09) | 5.29E-14 | (-0.04, -0.02) | 0.350 |
| Food focus | 0.54 (0.09) | 0.51 (0.08) | 1.39E-13 | (-0.04, -0.02) | 0.335 |
| Self-conscious | 0.83 (0.07) | 0.86 (0.09) | 6.30E-12 | (0.02, 0.03) | -0.320 |
| Health oriented | 0.93 (0.08) | 0.90 (0.13) | 1.98E-09 | (-0.04, -0.02) | 0.284 |
| Need curiosity | 0.88 (0.11) | 0.85 (0.14) | 5.23E-08 | (-0.04, -0.02) | 0.253 |
| Body focus | 0.87 (0.13) | 0.84 (0.16) | 3.73E-04 | (-0.04, -0.01) | 0.165 |
| Facet modesty | 0.63 (0.21) | 0.66 (0.21) | 5.87E-04 | (0.01, 0.05) | -0.157 |
| Facet imagination | 0.70 (0.14) | 0.73 (0.14) | 6.04E-04 | (0.01, 0.03) | -0.156 |
| Need ideal | 0.51 (0.19) | 0.48 (0.20) | 0.0010283 | (-0.05, -0.01) | 0.150 |
| Facet orderliness | 0.09 (0.08) | 0.06 (0.06) | 2.95E-24 | (-0.04, -0.03) | 0.463 |
| Religion oriented | 0.49 (0.11) | 0.46 (0.11) | 1.63E-07 | (-0.04, -0.02) | 0.240 |
| Empathetic | 0.41 (0.06) | 0.43 (0.07) | 4.25E-15 | (0.02, 0.03) | -0.365 |
| Facet cautiousness | 0.68 (0.15) | 0.66 (0.16) | 0.0006705 | (-0.04, -0.01) | 0.156 |
| Artistic | 0.67 (0.13) | 0.65 (0.14) | 0.0017123 | (-0.03, -0.01) | 0.144 |
| Facet gregariousness | 0.04 (0.06) | 0.02 (0.03) | 1.46E-31 | (-0.03, -0.02) | 0.525 |
| Value hedonism | 0.19 (0.08) | 0.21 (0.08) | 1.13E-10 | (0.02, 0.03) | -0.297 |
| Facet liberalism | 0.92 (0.09) | 0.94 (0.07) | 8.69E-11 | (0.02, 0.03) | -0.292 |
| Future | 0.78 (0.03) | 0.77 (0.03) | 6.74E-14 | (-0.01, -0.01) | 0.346 |
| Past | 0.71 (0.03) | 0.72 (0.03) | 5.89E-08 | (0.00, 0.01) | -0.248 |
| Generous | 0.42 (0.08) | 0.43 (0.08) | 6.53E-07 | (0.01, 0.03) | -0.229 |
| Present | 0.65 (0.03) | 0.66 (0.03) | 1.10E-04 | (0.00, 0.01) | -0.177 |
| Money oriented | 0.45 (0.07) | 0.46 (0.09) | 4.92E-04 | (0.01, 0.02) | -0.161 |
| Anxious | 0.81 (0.08) | 0.80 (0.08) | 0.001255 | (-0.02, -0.00) | 0.147 |
| Impulsive | 0.36 (0.08) | 0.35 (0.09) | 0.003861 | (-0.02, -0.00) | 0.132 |
| Facet intellect | 0.96 (0.04) | 0.97 (0.04) | 0.0192 | (0.00, 0.01) | -0.106 |
| Humble | 0.53 (0.11) | 0.54 (0.12) | 0.0321026 | (0.00, 0.02) | -0.099 |
| Big5 openness | 0.62 (0.22) | 0.61 (0.24) | 0.3208442 | (-0.03, 0.01) | 0.046 |
| Value self enhancement | 0.15 (0.10) | 0.14 (0.08) | 3.36E-04 | (-0.02, -0.01) | 0.162 |
| Need practicality | 0.21 (0.11) | 0.20 (0.10) | 0.0354413 | (-0.02, -0.00) | 0.095 |
| Openness | 0.46 (0.11) | 0.47 (0.10) | 0.0095124 | (0.00, 0.02) | -0.117 |
| Adjustment | 0.01 (0.01) | 0.01 (0.00) | 9.73E-09 | (-0.00, -0.00) | 0.247 |
| Netspeak focus | 0.28 (0.03) | 0.28 (0.03) | 0.0151397 | (0.00, 0.01) | -0.113 |

**Std Dev = Standard deviation*

**All scores are within (0, 1)*
